# Effects of topping and non-topping on growth-regulating hormones of flue-cured tobacco (*Nicotiana tabacum L*.)—a proteomic analysis

**DOI:** 10.3389/fpls.2023.1255252

**Published:** 2023-10-30

**Authors:** Kaiyuan Gu, Li-E. Yang, Ke Ren, Xianxue Luo, Xiao Qin, Michiel Op de Beeck, Conglian He, Li Jian, Yi Chen

**Affiliations:** ^1^ Agronomic Center, Yunnan Academy of Tobacco Agricultural Sciences, Kunming, Yunnan, China; ^2^ Biotechnology and Germplasm Resources Institute, Yunnan Academy of Agricultural Sciences, Kunming, Yunnan, China; ^3^ Raw material center, Hunan Zhangjiajie Municipal Tobacco Co., Zhangjiajie, Hunan, China; ^4^ Centre for Environmental and Climate Research, Lund University, Lund, Sweden; ^5^ Key Laboratory of Urban Environment and Health, Instituteo of Urban Enviroment, Chinense Academy of Sciences, Xiamen, Fujian, China

**Keywords:** tobacco, proteomics, leaves and roots, plant hormones, topping

## Abstract

**Introduction:**

Until now, the mechanism underlying the impact of topping on hormone regulation in tobacco plants remains unclear, and most studies investigating the hormone signaling pathways in plants rely on genes or transcriptional pathways.

**Methods:**

This study examines the regulatory mechanisms of hormones in the roots and leaves of tobacco plants with and without topping at the protein level.

**Results:**

The results demonstrate that, compared with non-topped plants, topping leads to a decrease in the levels of IAA (auxin), ABA (abscisic acid), and GA (gibberellin) hormones in the leaves, whereas the content of the JA (jasmonic acid) hormone increases. Furthermore, in the roots, topping results in an increase in the levels of IAA, ABA, and JA hormones, along with a decrease in GA content. In the leaves, a total of 258 significantly different proteins were identified before and after topping, with 128 proteins upregulated and 130 proteins downregulated. In the roots, there were 439 proteins with significantly different quantities before and after topping, consisting of 211 upregulated proteins and 228 downregulated proteins. Notably, these proteins were closely associated with the metabolic and biosynthetic pathways of secondary metabolites, as indicated by functional categorization.

**Conclusions:**

When integrating the hormone changes and the proteomics results, it is evident that topping leads to increased metabolic activity and enhanced hormone synthesis in the root system. This research provides a theoretical foundation for further investigations into the regulation and signaling mechanisms of hormones at the protein level before and after topping in plants.

## 1 Introduction

Tobacco is primarily cultivated for its leaves, with only a few fields dedicated to producing improved seeds. Once the flower buds appear, tobacco plants shift from vegetative growth to reproductive growth, diverting nutrients to their top buds. This change negatively impacts leaf growth and the concentration of internal compounds, resulting in reduced yield and quality of tobacco leaves. Topping, a method of removing the apical dominance of plants, plays a crucial role in regulating the nutrition of tobacco plants and enhancing the yield and quality of leaves. Recent research has revealed that topping inhibits the transfer of nutrients to reproductive organs, allowing more photosynthates to be distributed into the leaves (Baldwin et al., 1994; Baldwin, 2001; Li et al., 2007). This leads to increased accumulation of dry matter in the leaves, promotes yellowing, and encourages axillary bud growth in flue-cured tobacco leaves. Ultimately, topping improves both the yield and quality of flue-cured tobacco leaves (Baldwin et al., 1994. Li et al., 2007).

Topping is a common agronomic measure that significantly affects the yield and quality of various crops (Baldwin et al., 1994; Baldwin, 2001; Li et al., 2007). In recent years, studies on tobacco topping have found that topping can affect the source–sink relationship, hormone balance, and root development and ultimately promote the ripening and yellowing of tobacco leaves and improve the quality of tobacco leaves (Gravalos et al., 2019. Gršić et al., 2014. Qi et al., 2012). Singh et al. found that topping upregulated the gene expression of stress response, hormone metabolism, and secondary metabolism in tobacco roots but downregulated after exogenous hormone treatment (Singh et al., 2015). Studies also showed that topping could also increase the contents of gibberellin (GA3) and abscisic acid (ABA) in plant leaves and roots, thus improving the quality of tobacco leaves (Zou and Su, 2008). To sum up, the physiological response of tobacco to topping is inseparable from the signal regulation of hormones.

So far, most studies aimed at revealing hormone signaling pathways in plants have relied on genes or transcription pathways (Guillory and Bonhomme, 2021; Garmash, 2022). However, in the past decade, the MS-driven proteomics approach has become an increasingly popular tool in plant research because it reveals the specific mechanisms of plant hormone control, which largely occurs in the proteome level. In addition, there are few studies on the changes of plant endogenous hormones on protein levels before and after tobacco topping. We propose that the levels of certain hormones in tobacco leaves and roots undergo changes following topping, as compared with their pre-topping levels. Additionally, we anticipate variations in protein expression levels between tobacco leaves and roots. We measured the changes of hormone and protein expression levels in tobacco leaves and roots of flue-cured tobacco before and after topping. The objectives of our study are (i) to understand the changes of phytohormones in tobacco leaves and roots before and after topping and, (ii) by comparing the hormone change trend and protein differential expression of flue-cured tobacco before and after topping, to explore the hormone regulation mechanism and signal transduction mechanism of topping on maturation and senescence of flue-cured tobacco, so as to provide a theoretical basis for high-quality and high-yield cultivation of flue-cured tobacco.

## 2 Materials and methods

### 2.1 Study area and sample collection

The experiment was conducted in Jiuxi Tobacco base, Yunnan Province (102°38′13′′ E, 24°18′14′′ N, 1,730 m above sea level). The “cherry-red” tobacco YQZ18 was used in this study, which was provided by Zhongyan Tobacco Seed Co., Ltd., China. The rainfall is 773 mm, the number of sunshine hours is 1,641.69 h, and the annual average temperature is 15.6°C. The soil in the study area was paddy soil. The soil basic physical and chemical properties were as follows: pH 6.68, organic matter 30.2 g·kg^−1^, alkali hydrolyzed nitrogen 101.6 mg·kg^−1^, available potassium 147.9 mg·kg^−1^, and available phosphorus 24.2 mg·kg^−1^. The cultivation and management of flue-cured tobacco were carried out in accordance with the local standard B53/T 182.16-2006 “Technical Specification for Field Management of High quality flue-cured Tobacco in Yuxi”. Flue-cured tobacco seedlings were raised by a floating seedling technique on February 25. On April 20, flue-cured tobacco was transplanted to a flowerpot with 33 cm in diameter and 27 cm in height. The topping treatment was performed on June 15, and sample collections were conducted separately before topping (June 15) and 7 days after topping (June 22) from 8:30 to 9:30 in the morning. Samples were collected from tobacco leaves (five leaves from top to bottom, removing main veins and larger branches) and roots (root tip 3–5 cm), with three biological repeats in each treatment. Flue-cured tobacco plants with the same growth were selected for each repeat. Samples were stored in liquid nitrogen instantly and sent to Wuhan Jinkairui Bioengineering Co., Ltd. (http://www.genecreate.cn/) for further testing.

### 2.2 Determination of endogenous hormone in the tobacco plant and root

Tobacco plant endogenous hormones, including IAA, JA, GA, and ABA, were measured using enzyme-linked immunoassay (ELISA) (Hao et al., 2000). A 0.5-g sample of fresh tobacco leaf or root was taken from each treatment, quickly placed in liquid nitrogen, then moved to a freezer at −60°C for storage. The samples were mixed with 3–5 ml 80% methanol, ground into a homogenate in an ice bath, transferred to a centrifuge tube, shaken, placed at 4°C for 4 h, and then centrifuged at 16,000 × g for 15 min. The supernatant was collected and passed through a C18 pretreatment column. The collected supernatant was dried with nitrogen at 45°C for hormone determination.

### 2.3 Protein extraction, digestion, and desalting

The protein extraction, digestion, and desalting steps followed the methods as described in previous publications (Xie et al., 2016; Cai et al., 2018). A 100-μg portion of protein was extracted from each sample and subjected to trypsin digestion. The obtained solution was then diluted five times with 100 mM triethylamine borane (TEAB). Trypsin was added to the mixture in a 1:50 mass ratio in relation to the protein, and the enzymatic hydrolysis was allowed to occur overnight at 37°C. The resulting peptide segments were desalted using a C18 column (5 μm, 100 A, 4.6 × 250 mm), then frozen and vacuum-dried for further analysis.

### 2.4 iTRAQ labeling and fractionation

The peptides were dissolved in a 0.5-ml TEAB solution and then labeled according to the instructions provided by the iTRAQ-8 standard kit from SCIEX. After labeling, the samples were mixed together. The mixed peptides were then separated using the UltiMate 3000 HPLC system from Thermo Dionex, USA. This separation was carried out using a Durashell C18 column (5 μm, 4.6 × 250 mm). The separation process involved increasing the concentration of ACN under alkaline conditions, with a flow rate of 1 ml/min. Each minute, one tube was collected. In total, 42 secondary fractions were collected and later merged into 12 components. These components were desalted using a Strata-X column and dried under vacuum.

### 2.5 LC-MS/MS analysis

The LC-MS/MS analysis was performed on an AB Sciex nanoLC-MS/MS (TripleTOF 5600 Plus) system. The polypeptide solution was added to the C18 capture column (5 μm, 100 μm × 20 mm), and gradient elution was carried out on the C18 analytical column (3 μm, 75 μm × 150 mm) at a 90-min time gradient and 300 nl/min flow rate. The two mobile phases are respectively mobile phase A (2% acetonitrile/0.1% formic acid/98% H_2_O) and mobile phase B (98% acetonitrile/0.1% formic acid/2% H_2_O). For information-dependent acquisition (IDA), the first-order mass spectrogram was scanned with 250-m/s ion accumulation time, and the second-order mass spectrogram of 30 precursor ions was collected with 50-ms ion accumulation time. The MS1 spectra were collected in the range of 350–1,500 m/z, and the MS2 spectra were collected in the range of 100–1500 m/z. The precursor ions were excluded from reselection for 15 s.

### 2.6 Protein identification and quantification

The initial MS/MS data were entered into ProteinPilot Software v4.5 from Thermo Fisher Scientific Inc. (USA), for the purpose of data analysis. The combination of ProteinPilot’s Paragon database search algorithm (ProteinPilot Software v4.5, Thermo Fisher Scientific Inc. (USA)) and non-linear fitting methods were utilized to determine the false discovery rate (FDR) for both peptide identification and quantification. In order to minimize the FDR, a protein identification threshold was implemented, requiring a confidence value of 95 (equivalent to the confidence value “unused ProtScore” 1.3 in ProteinPilot software). At least one unique peptide was necessary for protein identification. Proteins exhibiting a |log2 fold-change| > 1 were categorized as DEPs.

### 2.7 Bioinformatics and annotations

In order to understand the biological and functional characteristics of all the proteins that were detected, the protein sequences were matched with Gene Ontology Terms (http://geneontology.org/). Initially, a homology search was conducted for all the identified sequences using a localized NCBI blast program against the NCBI nr data base. The reference database was PR1-20060018-uniprot-Nicotiana_tabacum.fasta. The proteins were annotated with KEGG using the Kyoto Encyclopedia of Genes and Genomes (KEGG) online service tool (available at http://www.genome.jp/kaas-bin/kaas_main).

### 2.8 Targeted protein quantification by parallel reaction monitoring (PRM)

The process of extracting protein and digesting it with trypsin was carried out following the previously outlined methods. Afterward, targeted MS analysis using PRM was performed on a TripleTOF 5600 LC-MS/MS system (AB Sciex, Massachusetts, USA). To collect PRM data, an inclusion list containing optimized peptide sequences is created, and the mass spectrometer sequentially selects each peptide on the list to be fragmented and analyzed for its secondary ion spectrum. The peptide samples obtained through enzymatic hydrolysis were combined and subsequently analyzed using mass spectrometry data-dependent acquisition (DDA) detection. The identification of proteins was accomplished by employing the ProteinPilot software, following which the spectrum library was established by introducing the Skyline software. The proteins to be validated through PRM were brought into the Skyline software, where the peptides to be used for quantifying the proteins were chosen based on their ion signals in the spectra library. The process of developing the PRM detection method involved exporting a list of peptides with their corresponding m/z values and retention times from Skyline, which was then imported into the MS control software Analyst. Then, the PRM technique was employed to conduct PRM data capture on the blended samples, in order to fine-tune and enhance the PRM data acquisition approach resulting in a definitive PRM methodology for gathering sample data. For every sample, the PRM mass spectrometry method was utilized to perform data acquisition in an optimized manner. The quadrupole was utilized to choose each precursor ion, which was then fragmented. Following the fragmentation, all of the fragment ions were quantified in the TOF mass analyzer. Performing a “blank” run to wash the column between consecutive samples was necessary in order to prevent protein carryover. The acquired data were processed using Skyline software, and the quantification outcomes were meticulously examined for each peptide that belonged to the targeted proteins. Extracting and analyzing PRM spectrum files enabled the acquisition of quantitative data regarding the desired protein (Luczak et al., 1997; Vidova and Spacil, 2017; Wang et al., 2018).

### 2.9 Data analysis

The KEGG online service tool (http://www.genome.jp/kaas-bin/kaas_main) was used to annotate the protein with KEGG. Subsequently, the differential proteins were enriched in the corresponding pathway using the KEGG mapper. The heat map was generated using the pheatmap package in RStudio (R 4.1.2) software, whereas the volcano plot and bubble diagram were generated using the ggplot2 package. The data of endogenous hormones were analyzed by SPSS 22.0 (SPSS Institute Inc.) and Origin 8.0 (Origin Lab).

## 3 Results

### 3.1 Plant type and root morphology of flue-cured tobacco before and after topping

**Figure 1:**
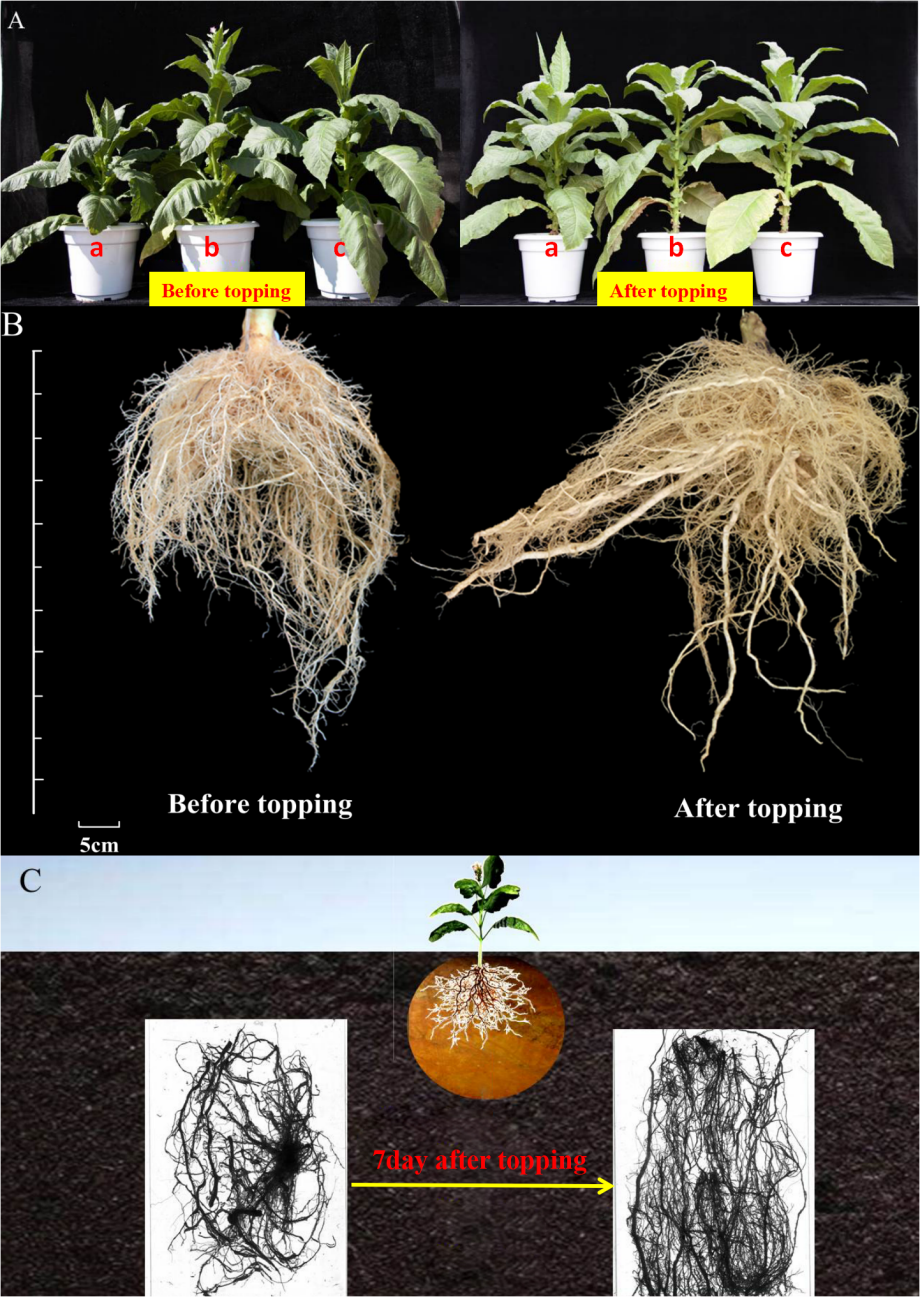
Plant type and root morphology of flue-cured tobacco before and after topping. **(A)** Comparison of the changes of leaf phenotype and plant type of tobacco before and after topping; **(B)** comparison of morphological changes of tobacco roots before and after topping; **(C)** scanning images of tobacco roots before and after topping.

After topping, the flue-cured tobacco plant type changes from a tower type to a tube type, and the tobacco leaves fall yellow from bottom to top one by one, the yellowing fall is fast, the layer yellow fall is obvious, and the leaves gradually mature (**Figure 1A**). The appearance of the root system of flue-cured tobacco before and after topping is shown in the figure (**Figures 1B, C**). After topping, the length of the root system of the flue-cured tobacco increases, and the number of lateral roots also increases.

### 3.2 Changes of endogenous hormones in tobacco plants before and after topping

**Figure 2:**
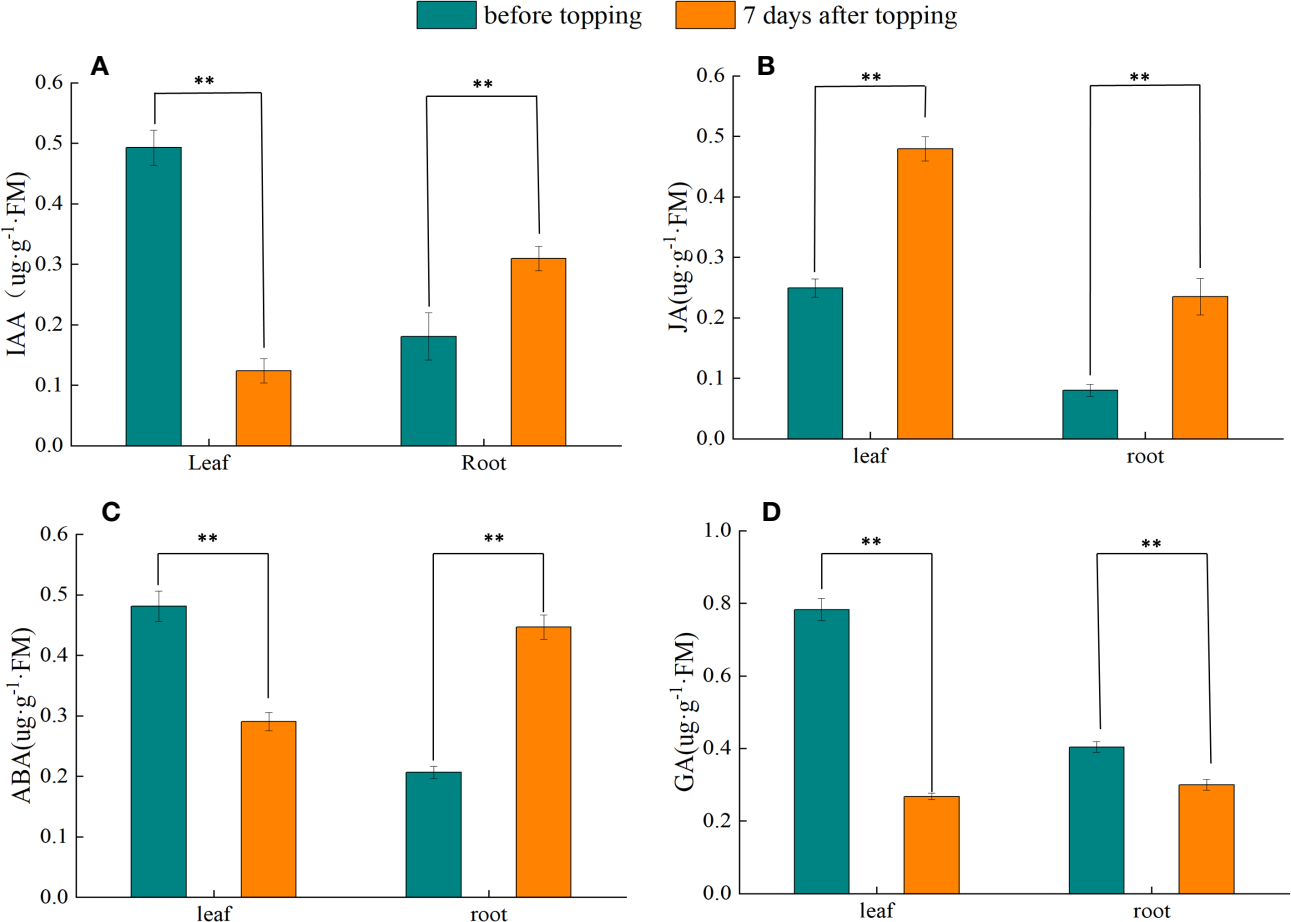
Changes of endogenous hormones in tobacco plants before and after topping. **(A)** IAA; **(B)** JA; **(C)** ABA; **(D)** GA. The data were tested by Duncan’s multiple-range test. “**” indicates a significant difference at the α = 0.01 level between the two groups of data. Data represent means ± standard error (n = 3) .

Seven days before topping, the IAA content of tobacco leaves was significantly higher than that of tobacco leaves after topping, whereas the IAA content of roots was significantly lower than that of roots after topping (**Figure 2A**). In the 7 days before topping, the JA content in tobacco leaves was significantly lower than that in tobacco leaves 7 days after topping, and the root JA content also showed an upward trend after topping (**Figure 2B**). Before and after topping, ABA in tobacco leaves showed a significant decreasing trend, whereas root ABA showed an increasing trend (**Figure 2C**). Before and after topping, the content of GA in tobacco leaves and roots showed a significant downward trend (**Figure 2D**). In summary, the topping treatment can significantly change the content of plant hormones in tobacco plants, thereby regulating the growth and development of tobacco plants.

### 3.3 Proteomics study of tobacco leaf and root system before and after topping

**Figure 3:**
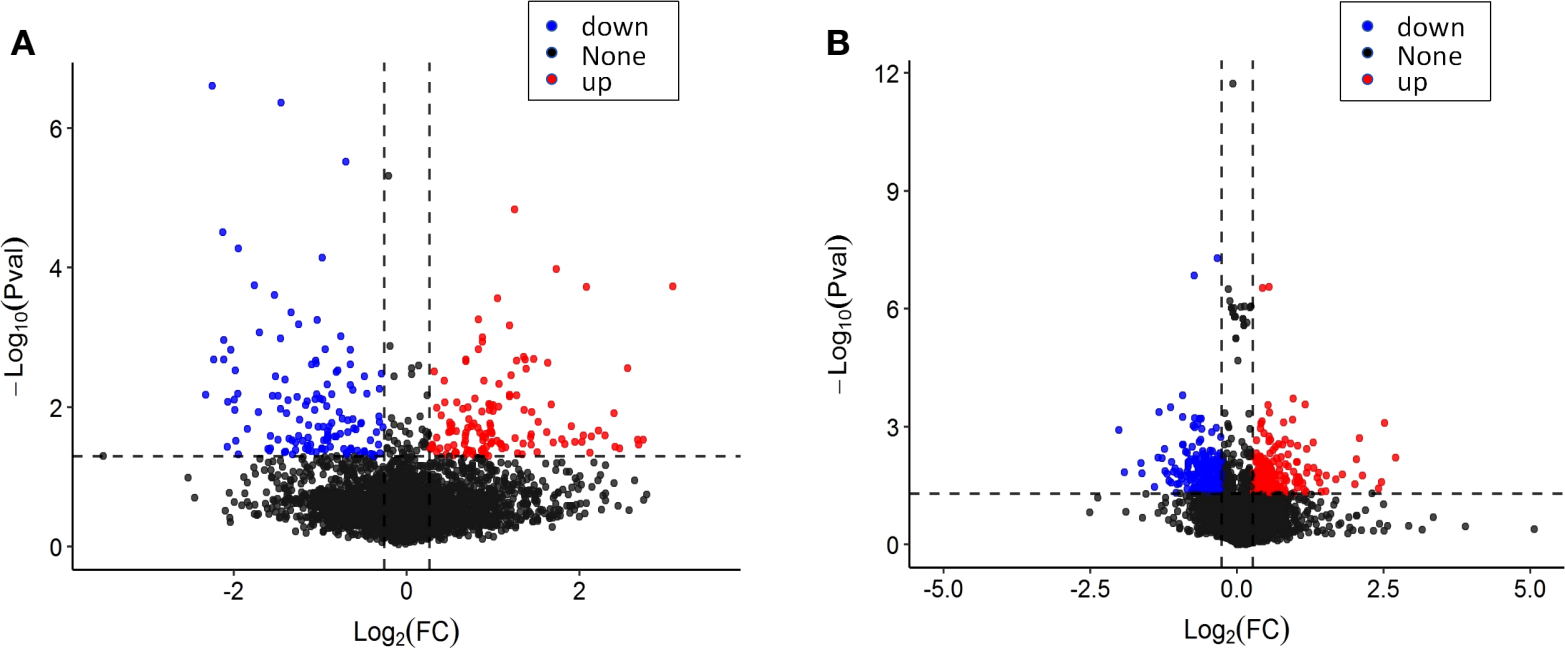
Protein expression of tobacco leaves and roots before and after topping. **(A)** Protein expression of tobacco leaves; **(B)** protein expression of tobacco roots.

In the proteomic analysis of tobacco leaves before and after topping, a total of 4,720 proteins were detected. Comparing the tobacco leaves before and after topping, there were 258 significantly different expressed proteins (DEPs), of which 128 were upregulated and 130 were downregulated (**Figure 3A**). In the proteomic analysis of the root system before and after topping, a total of 7,902 proteins were detected. Comparing the root system before and after topping, it was found that there were 439 significantly DEPs, of which 211 DEPs were upregulated and 228 different proteins were downregulated (**Figure 3B**). The comparison between tobacco leaves and roots showed that the DEPs in roots were more numerous than those of in tobacco leaves.

**Figure 4:**
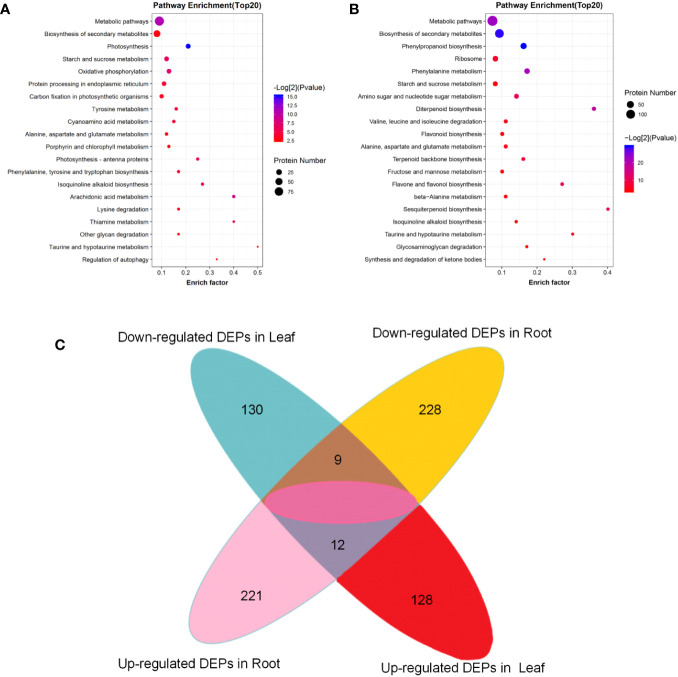
KEGG pathway enrichment of the DEPs. **(A)** KEGG pathway enrichment of the DEPs in tobacco leaf; **(B)** KEGG pathway enrichment of the DEPs in tobacco root; **(C)** Venn diagram of DEPs in tobacco leaf and root system before and after topping.

In the tobacco leaf comparison group before and after topping, the DEPs were mainly involved in six metabolic processes, five photosynthesis-related processes, three photoreaction processes, and six other biological processes (**Figure 4A**). In Molecular Function, the first 20 molecular functions involved in DEPs included nine binding functions and 11 catalytic activity functions (**Supplementary Figure 1**). In the cellular component, DEPs were mainly involved in the formation of four organelle structures, five thylakoid structures, eight photosynthetic system structures, and three other structures (**Supplementary Figure 1**). In the root comparison group before and after topping, the DEPs were mainly involved in two stress processes, six metabolic processes, five catabolic processes, six ion migration, and one other biological process (**Supplementary Figure 1**). In molecular function, the DEPs mainly have eight binding functions and 12 catalytically active functions. In the cellular component, the DEPs are mainly involved in the formation of plasma membrane structure, vesicle structure, and other structures (**Supplementary Figure 1**).

In the KEGG enrichment analysis of DEPs in tobacco leaves before and after topping, the DEPs were significantly enriched in metabolic pathways (93), biosynthesis of secondary metabolites (44), photosynthesis (16), starch and sucrose metabolism (14), and other metabolic pathways, including six pathways related to amino acid metabolism (**Figure 4A**). In the KEGG enrichment analysis of root DEPs before and after topping, the DEPs were significantly enriched in metabolic pathways (134), biosynthesis of secondary metabolites (94), phenylpropanoid biosynthesis (35), ribosome (25), amino acid metabolism-related pathways, and biological synthetic pathways, and other metabolic pathways (**Figure 4B**). A total of 21 common differential proteins were screened from flue-cured tobacco leaves and roots after topping, including 12 upregulated proteins and 9 downregulated proteins.

The top 20 pathway enrichment terms (*p < 0.05*) are displayed, the pathway name (y-axis) and rich factor (x-axis). The color of the dot represents a p-value, and the size of the dot indicates the number of DEPs enriched in the pathway. The rich factor is the ratio of the number of DEPs in one pathway to the number of all background proteins in the same pathway. The larger the rich factor, the more significant the enrichment level of the DEPs in this pathway.

**Figure 5:**
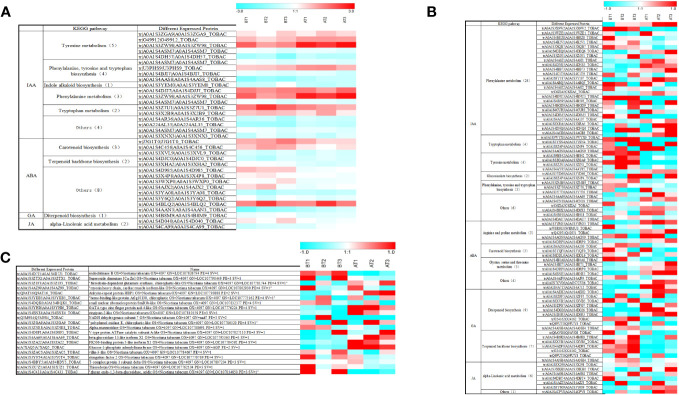
Heat diagram of the relative expression matrix of the DEPs. **(A)** Heat diagram of the relative expression matrix of the two groups of differential proteins. **(B)** Heat diagram of the relative expression matrix of tobacco leaf-related hormone. **(C)** Heat diagram of the relative expression matrix of tobacco root-related hormone. The color in the figure indicates the relative expression level of the protein in the sample. The red color indicates that the protein has a higher expression level in the sample, and the blue color represents a lower expression level. Color bars represent specific expression abundance.

There were 21 DEPs between the two groups before and after topping (**Figure 4C**). These DEPs are mainly involved in biological processes such as metabolic processes, cellular processes, and stress responses. Among them, 12 DEPs were related to stress response, and five DEPs were involved in pathways related to plant endogenous hormones, including GATA type zinc finger protein asd-4-like, polyphenol oxidase E, chloroplastic-like, and beta-glucosidase 13-like isoform X1, FK506-binding protein 5-like isoform X1, and acidic endo-1,3-beta-glucosidase (**Figure 5C**).

**Figure 6:**
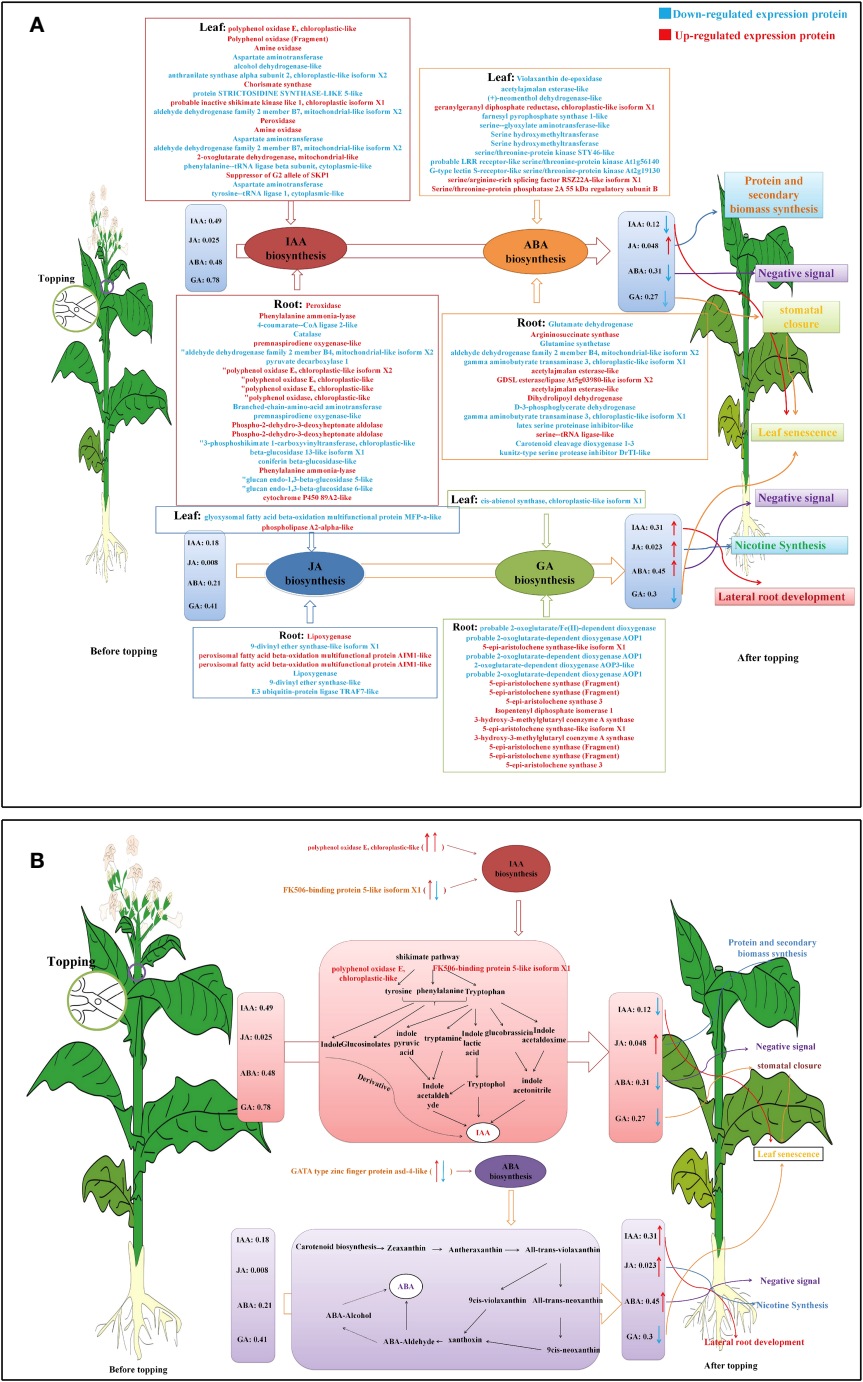
Hormone biosynthesis pathways in tobacco leaf and root. **(A)** DEPs related to four hormone biosynthesis pathways. **(B)** DEPs related to the IAA and ABA biosynthesis pathway in tobacco leaf and root. The increased DEPs were marked with an upward red arrow and red colored words, and the decreased DEPs were marked with a downward green arrow and green-colored words. The arrow on the left indicates the DEPs in leaf, and the arrow on the right indicates the DEPs in root.

In the proteomic study of tobacco leaves before and after topping, the pathways related to changes in leaf IAA included tyrosine metabolism (5), phenylalanine, tyrosine and tryptophan biosynthesis (4), indole alkaloid biosynthesis (1), tryptophan metabolism (2), and other pathways (4). Metabolic pathways associated with changes in ABA were carotenoid biosynthesis (3), terpenoid backbone biosynthesis (2), and others (8). Metabolic pathways associated with changes in GA included diterpenoid biosynthesis (1). Metabolic pathways associated with changes in JA included alpha-linolenic acid metabolism (2) (**Figures 5A**, **6**).

The pathways related to changes in root IAA included phenylalanine metabolism (24), tryptophan metabolism (4), tyrosine metabolism (4), glucosinolate biosynthesis (4), phenylalanine, tyrosine, and tryptophan biosynthesis (3), and other pathways (6). The pathways related to changes in ABA included arginine and proline metabolism (5), carotenoid biosynthesis (3), glycine, serine, and threonine metabolism (3), and other pathways (4). The pathways related to GA changes included diterpenoid biosynthesis (9) and terpenoid backbone biosynthesis (7). The pathways related to changes in JA include alpha-linolenic acid metabolism (6) and other pathways (1) (**Figures 5B**, **6**).

**Figure 7:**
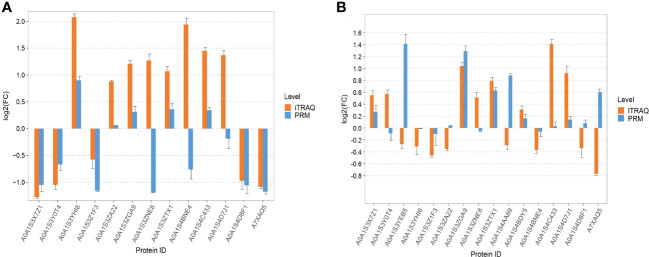
Parallel reaction monitoring verification. **(A)** The PRM verification of tobacco leaves. **(B)** The PRM verification of tobacco roots.

According to the results of iTRAQ, PRM verified 13 DEP_S_ in tobacco leaves and 16 DEPs in tobacco roots. In the PRM verification of tobacco DEPs, the results of 10 proteins were consistent with those of iTRAQ, of which five proteins were upregulated, five proteins were downregulated, and the results of the other three proteins were opposite to those of iTRAQ (**Figure 7A**). In the PRM verification of root DEPs, the results of nine proteins were consistent with those of iTRAQ, of which six proteins were upregulated, three proteins were downregulated, and seven proteins were contrary to those of iTRAQ (**Figure 7B**).

## 4 Discussion

### 4.1 Phytohormone and tobacco plant phenotype

Topping is an important technical link in flue-cured tobacco production, which can break the balance between reproductive growth and vegetative growth of flue-cured tobacco, make more nutrients into tobacco leaves, promote the growth and development of tobacco roots, increase the accumulation of dry matter in tobacco leaves, and increase the effective area of upper tobacco leaves (Li et al., 2007; Baldwin et al., 1994). The phenotypic results of this treatment in current studies confirm this conclusion.

Previous studies have shown that plant hormones, as important tracer signal molecules during stress resistance, can regulate the stress response and growth and development of plants (Davies, 2010; Fahad et al., 2015). After topping, the content of IAA in tobacco leaves showed a downward trend, whereas the synthesis of IAA in roots increased significantly, because IAA was mainly synthesized at the top of the plant, and after topping, the endogenous auxin synthesis at the tip was missing, so that the IAA synthesized by apical meristem was synthesized in the root tip meristem, thus promoting the development of tobacco lateral roots (Shoji et al., 2000). Therefore, the root activity was enhanced and the ability of nutrient absorption was enhanced.

Jasmonic acid (JA) is an oxidized lipid derived from α-linolenic acid, which regulates a series of plant growth and development processes. Previous studies have shown that jasmonic acid is directly related to plant resistance to mechanical injury and disease resistance (Wasternack, 2007). JA is an essential component of signal transduction that drives plant defense gene expression in stress response (Elliot, 1975). When plants are stressed, jasmonic acid content increases sharply, inducing the expression of specific genes. Promote the synthesis of protein and secondary substances, so as to improve the stress resistance of plants (Wang et al., 2009). After topping, the content of JA in tobacco leaves and roots increased significantly, which may be because the mechanical damage caused by topping activated the stress resistance response of flue-cured tobacco. In addition, Van der Graaff et al. (2006) proved that the expression of jasmonic acid synthesis-related genes increased during Arabidopsis leaf senescence, and the content of jasmonic acid increased gradually during leaf senescence, which also indicated that topping promoted the maturation and senescence of flue-cured tobacco leaves.

Liang et al. found that rice OsNAP transcription factors are induced by abscisic acid and act (ABA) directly on abscisic acid synthesis and senescence-related genes, which proves the positive regulatory effect of abscisic acid on senescence (Liang et al., 2014). This study found that after topping, the ABA content of tobacco leaves decreased and leaves yellowed and senescent, which further proved the positive regulatory role of abscisic acid in flue-cured tobacco leaf senescence, whereas the proportion of ABA in roots increased significantly after topping. It was found that the decrease of IAA content in flue-cured tobacco leaves as a negative signal transported downward along the xylem as a negative signal would lead to an increase in the content of second messenger ABA in roots. This experiment is consistent with the results of this study. Some scholars believe that the effect of IAA is opposite to that of ABA, and that the increase of ABA content is beneficial to root development (Zhang et al., 2020), which echoes in phenotype.

Under stress, GA can stimulate stomatal closure and promote the transport of photosynthetic products to developing seeds (Zhou and Leul, 1998; Zhang et al., 2012). However, due to a lack of reproductive growth after topping, more photosynthetic products are transported to leaves. In addition, auxin and gibberellin could delay leaf senescence. After topping, the contents of auxin and gibberellin GA decreased significantly, which accelerated the senescence process of tobacco leaves (Umemura et al., 2023), which may be the reason why tobacco leaves gradually yellowed from the bottom to the top.

### 4.2 Hormone synthesis pathway and signal transduction pathway

IAA is converted from tryptophan. The common synthetic pathways are the indole pyruvate pathway, tryptamine pathway, indole acetonitrile pathway, and indoleacetamide pathway (Cohen et al., 2003; Sauer et al., 2013). Tryptophan is an important prerequisite for plant synthesis of IAA. In this study, after topping, the expression of several DEPs involved in tyrosine metabolism and phenylalanine metabolism in tobacco leaves was upregulated, whereas the expression of DEPs related to tryptophan biosynthesis and indole alkaloid biosynthesis was downregulated, indicating that the metabolism of tyrosine and phenylalanine in tobacco leaves increased after topping, whereas the substrate of tryptophan synthesis and indole synthesis decreased, resulting in the decrease of auxin content in tobacco leaves. Among the related pathways of auxin synthesis in tobacco roots, there are a large number of DEPs involved in tyrosine metabolism and phenylalanine, tyrosine, and tryptophan biosynthesis and downregulated expression of DEPs involved in tryptophan metabolism and glucosinolate biosynthesis. IAA synthesis in plants is mainly a tryptophan-dependent pathway, whereas the tryptophan synthesis pathway is inhibited after topping (Duca and Glick, 2020). The synthetic pathway of root auxin after topping may be the indole pyruvate and tryptamine pathways.

The synthesis of abscisic acid in plants is related to the terpenoid pathway and carotenoid pathway, in which the carotenoid pathway is the main pathway of ABA biosynthesis (Bartley et al., 1994; Milborrow, 2001; Cutlers, 2007). In this study, the metabolic pathways related to ABA synthesis in tobacco encompass processes such as carotenoid biosynthesis, terpenoid trunk biosynthesis, and other associated biochemical pathways. In this study, DEPs in the carotenoid biosynthesis pathway in tobacco leaves was significantly downregulated, whereas DEPs in the root carotenoid biosynthesis pathway was significantly upregulated, which was consistent with the change of hormone content in flue-cured tobacco, which further explained the decrease of ABA content in flue-cured tobacco leaves and the increase of root ABA content after topping.

In the process of GA biosynthesis, the diterpenoid biosynthetic pathway is the prerequisite for GA synthesis (Thomas and Hedden, 2006). In this study, the metabolic pathway related to tobacco GA is the diterpenoid biosynthesis, and the DEPs involved in diterpenoid biosynthesis are downregulated, indicating that the diterpenoid biosynthetic pathway of tobacco leaves was weakened after topping, which leads to the decrease of GA content in tobacco. These DEPs involved in the biosynthesis of diterpenoids may be involved in regulating the biosynthesis of GA. In DEPs involved in diterpene biosynthesis, the number of DEPs was upregulated. Among the DEPs involved in diterpenoid biosynthesis, the number of upregulated DEPs was higher than that of downregulated DEPs. The seven DEPs involved in the biosynthesis of the terpenoid backbone all showed upregulation, and the decrease in GA content in roots after topping may be the result of the weakened diterpenoid biosynthetic pathway.

The JA signaling pathway plays an important role in the response mechanism of biotic and abiotic stresses. The synthesis of JA begins with linolenic acid and some intermediate metabolites (Luczak et al., 1997). In this study, the metabolic pathway related to tobacco JA is the α-linolenic acid metabolic pathway, and the metabolic pathway related to rhizosphere JA includes the α-linolenic acid metabolic pathway and other metabolic pathways. In tobacco leaves and roots, several differential proteins involved in α-linolenic acid metabolism were significantly upregulated, which indicated that the mechanical damage of topping activated the α-linolenic acid metabolic pathway in tobacco leaves and roots and finally promoted the biosynthesis of JA (Pirrello et al., 2014; Wasternack and Song, 2017).

### 4.3 Hormones and proteins

Among the 21 common DEPs in tobacco leaves and roots before and after topping, 12 DEPs have stress response functions, and 4 DEPs were related to plant hormones. The acidic glucan endo-1,3-beta-glucosidaseis upregulated after topping. Studies have shown that acidic glucan endo-1,3-beta-glucosidaseis a hydrolase, which plays an important role in plant disease resistance and can induce the accumulation of enzymes related to plant disease resistance (Boller, 1987). When tobacco leaves are infected with tobacco mosaic virus (TMV) and induced by ethylene, the expression of β-1,3-glucanase will increase (Felix and Meins, 1987; Legrand et al., 1987; Vögeli-Lange et al., 1988). The expression of acidic glucan endo-1,3-beta-glucosidase increased after topping, indicating that the topping of tobacco plants promoted the expression of resistance-related proteins, which can enhance the disease resistance of the plant and is more beneficial to healthy growth of the leaves.

The protein GATA-type zinc finger protein asd-4-like is involved in the regulation of ABA biosynthesis and zinc finger protein may be involved in GA biosynthesis (Liu et al., 2010). Recent studies have provided emerging evidence that plant GATA zinc finger transcription factors also play significant roles in developmental control and responses to the environment (Zhang et al., 2015). The identification of the GATA23 transcription factor represents a first step toward the elucidation of the mechanisms that plants use to control lateral root spacing and guarantee an optimal distribution of new organs during the colonization of soil (De Rybel et al., 2010). GATA-type zinc finger protein asd-4-like was upregulated in tobacco leaves and downregulated in roots, whereas the content of ABA and GA in tobacco leaves decreased and ABA increased in roots; GA showed a decreasing trend in root. It shows that GATA-type zinc finger protein asd-4-like has a greater impact on the synthesis of gibberellin in roots after topping.

The polyphenol oxidase E, chloroplastic-like participates in the pigment biosynthetic process with the function of catalyzing the oxidation of mono- and o-diphenols to o-diquinones. It also participates in the metabolism of tyrosine, which is the substrate for the synthesis of indole and aromatic glucosinolates. Studies have shown that the activation of polyphenol oxidase leads to the oxidation of phenolic compounds, consequently enhancing the resistance, thus playing a defensive role (Nawrot et al., 2016). Polyphenol oxidase E, chloroplastic-like was upregulated in the tobacco leaves and roots after topping. The IAA content of the tobacco leaves decreased, whereas the IAA content in the roots increased. It may be because the protein plays a major role in the synthesis of IAA in roots. It has no obvious effect in the synthesis of IAA in tobacco leaves. After the topping, the IAA synthesis center shifted from the above-ground part to the underground part, the IAA synthesis in tobacco leaf was reduced, and the IAA synthesis in the root system was increased, so polyphenol oxidase E, chloroplastic-like played a better role in root system.

In the biosynthetic pathway of phenylalanine, tyrosine, and tryptophan, the three key enzymes involved in this pathway are anthranilate phosphoribosyl transferase (Trp D), tyrosine aminotransferase (TAT), and aspartame glycine aminotransferase (GOT1). FK506-binding protein 5-like isoform X1 was upregulated in the topped tobacco leaves but downregulated in the roots. The auxin content in the tobacco leaves decreased, whereas the auxin content in the roots increased. This may be because the protein was upregulated in tobacco leaves and downregulated in roots, which leads to increased metabolism of tyrosine, glycine, serine, threonine, and phenylalanine in tobacco leaves but decreases in the roots, accumulating enough IAA synthetic substrate in roots, resulting in an increase of IAA content in the root system after topping.

By studying the changing trend of hormones and proteomic analysis of tobacco after topping, we can thoroughly explain the interaction mechanism between hormones and the proteome of flue-cured tobacco from both a plant physiology and molecular perspective. From a biological point of view, this study is helpful to reveal the regulatory network of plant senescence under hormone regulation, and at the same time, it also provides a scientific theoretical basis for the use of suitable hormones to regulate the process of plant maturation and senescence, and to explore new ways of plant management, to provide a scientific theoretical basis for achieving high quality, high yield, and high efficiency of plants.

## 5 Conclusion

After topping, the contents of IAA, ABA, and GA in tobacco leaves decreased, and the content of JA increased; the contents of IAA, JA, and ABA in roots increased, and the content of GA decreased. In the proteomic study, the DEPs related to endogenous hormone synthesis in the root system were significantly more abundant than those in tobacco leaves. Combined with the results of hormone changes and proteomics, topping led to the increase of root metabolic activity and hormone synthesis. This study laid a theoretical foundation for the regulation and signal transmission mechanism of hormones at the protein level before and after topping.

## Data availability statement

Mass Spectrometry Proteomics data has been stored in the ProteomeXchange complex through the Pride partner repository with dataset identifier PXD043495.

## Author contributions

KG: Writing – original draft. L-EY: Data curation, Writing – original draft. KR: Methodology, Writing – review & editing. XL: Methodology, Writing – review & editing. XQ: Project administration, Writing – original draft. MOdB: Writing – review & editing. CH: Conceptualization, Writing – review & editing. LJ: Writing – review & editing. YC: Supervision, Writing – review & editing.

## Supplementary material

Supplementary Figure 1 GO enrichment Analysis of DEPs; **(A–C)** GO enrichment Analysis of DEPs in tobacco leaf; **(D–F)**: GO enrichment Analysis of DEPs in tobacco root.
