# Supplementary figures and images for: Effects of topping and non-topping on growth-regulating hormones of flue-cured tobacco (Nicotiana tabacum L.)—a proteomic analysis
Source: Front Plant Sci. 2023 Oct 30;14:1255252. doi: 10.3389/fpls.2023.1255252 (PMC10643189; doi:10.3389/fpls.2023.1255252)

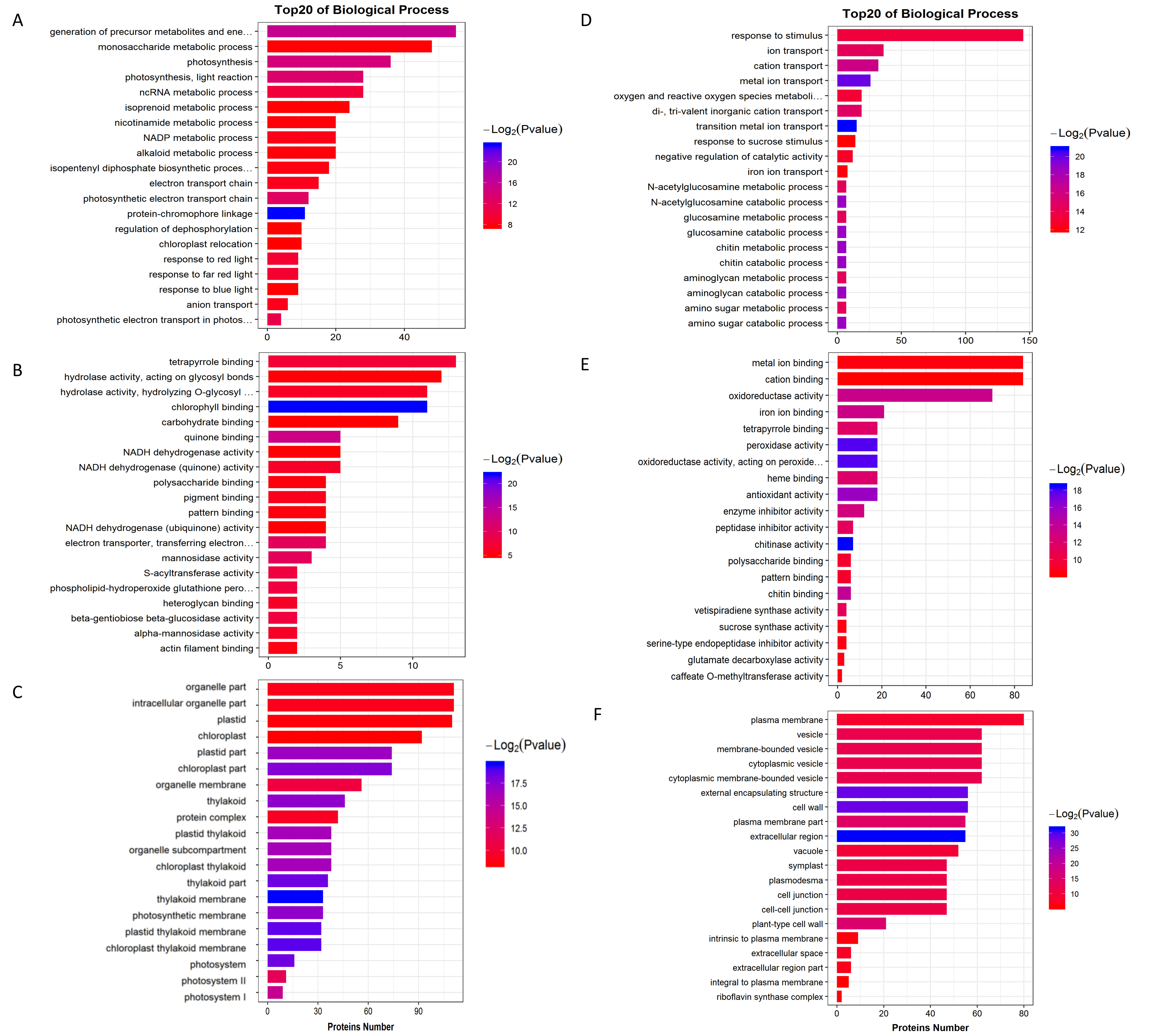

Supplement: Supplementary Figure 1 — GO enrichment Analysis of DEPs; (A–C) GO enrichment Analysis of DEPs in tobacco leaf; (D–F): GO enrichment Analysis of DEPs in tobacco root. [file Image_1.jpeg]
